# Trap-Filling of ZnO Buffer Layer for Improved Efficiencies of Organic Solar Cells

**DOI:** 10.3389/fchem.2020.00399

**Published:** 2020-05-26

**Authors:** Mingguang Li, Jing Li, Longsheng Yu, Ying Zhang, Yizhong Dai, Runfeng Chen, Wei Huang

**Affiliations:** ^1^Key Laboratory for Organic Electronics and Information Displays & Jiangsu Key Laboratory for Biosensors, Institute of Advanced Materials (IAM), Jiangsu National Synergetic Innovation Center for Advanced Materials (SICAM), Nanjing University of Posts & Telecommunications, Nanjing, China; ^2^Institute of Flexible Electronics, Northwestern Polytechnical University, Xi'an, China

**Keywords:** ZnO, cathode buffer layers, organic solar cells, trap-filling, organic–inorganic hybrid film

## Abstract

Trap-assisted recombination loss in the cathode buffer layers (CBLs) is detrimental to the electron extraction process and severely restricts the power conversion efficiencies (PCEs) of organic solar cells (OSCs). Herein, a novel organic–inorganic hybrid film composed of zinc oxide (ZnO) and 2,3,5,6-tetrafluoro-7,7,8, 8-tetracyanoquinodimethane (F4TCNQ) is designed to fill the intrinsic charge traps of ZnO-based CBLs by doping F4TCNQ for high-performance inverted OSCs. Thus, constructed ZnO:F4TCNQ hybrid film exhibits enhanced surface hydrophobicity and adjustable energy levels, providing favorable interfacial condition for electron extraction process. Consequently, trap-assisted recombination loss in the CBLs was efficiently suppressed, leading to the significantly improved fill factor and PCEs of both fullerene- and non-fullerene-based OSCs using the ZnO:F4TCNQ hybrid CBLs. This work illustrates a convenient organic acceptor doping approach to suppress the internal charge traps of traditional inorganic CBLs, which will shed new light on the fabrication of high-performance CBLs with facile electron extraction processes in inverted OSC devices.

## Introduction

Bulk heterojunction (BHJ) organic solar cells (OSCs) have attracted extensive attention due to their intrinsic merits of light weight, high mechanical flexibility, and easy processing (Li et al., 2012; Yan et al., 2018). In the construction of high-performance OSC devices, both the regulation of photoactive layers and interface engineering of the integrated functional layers plays important roles in achieving high power conversion efficiencies (PCEs) (Liu et al., 2016, 2018; Liu T. et al., 2017). As for a specified photoactive layer, interfacial modification by using an efficient cathode buffer layer (CBL) has been widely applied to further optimize the device structure and improve the device efficiency (Yin et al., 2016).

Among various interfacial materials, the zinc oxide (ZnO) film inserted between organic photoactive layer and inorganic electrode is one of the most widely used CBLs in the inverted OSCs, exhibiting many inherent advantages, including high electron mobility, high optical transparency, tuneable electronic properties, ease of preparation, and low toxicity (Hewlett and McLachlan, 2016). Moreover, a simple and extensively investigated sol-gel method can be applied to fabricate ZnO-based CBLs at a relatively mild condition (Kamalasanan and Chandra, 1996). However, the low-temperature solution-processed ZnO films generally have high density of surface and bulk defects, which would introduce undesired intra-gap energy levels and thus suppress carrier transport processes in the CBLs. These intrinsic defects in ZnO lattice mainly comprise oxygen vacancies (V_O_), oxygen interstitials (O_i_), zinc vacancies (V_Zn_), zinc interstitials (Zn_i_), etc. (Spencer et al., 2012). Plenty of defects acting as charge recombination centers will reduce the charge collection efficiency, which is an obstacle to the application of ZnO material in the field of optoelectronic devices (Sun et al., 2011; Lee et al., 2019).

In view of the above adverse issues, there have been a general consensus to optimize charge transporting properties of ZnO-based CBLs by passivating the defect sites (Hu et al., 2015; Cai et al., 2017; Li et al., 2017; Wang et al., 2019). For example, a high-performance fullerene-free OSC with efficiency over 12% was reported by employing ethylene diamine tetraacetic acid (EDTA)/ZnO hybrid interlayer. The introduced EDTA efficiently passivizes defects in ZnO film due to its chelation function, leading to balanced hole and electron mobility (Li et al., 2017). Besides, amphiphilic fullerenes/ZnO hybrids were also used as novel CBLs to passivate the defects of ZnO and the corresponding efficiency of the OSC based on thieno[3,4-b]-thiophene/benzodithiophene (PTB7):PC_71_BM was up to 8.0% (Hu et al., 2015). Hybrid CBL consisting of ZnO and poly(2-ethyl-2-oxazoline) (PEOz) was also constructed and applied to the inverted non-fullerene OSCs. Due to the reduced oxygen deficiency of ZnO by the introduced PEOz, an improved PCE over 12% was achieved (Seo et al., 2018). Additionally, various other organic materials such as polyethylenimine (PEI) (Liu C. et al., 2017), fullerene derivatives (Liao et al., 2014; Hu et al., 2016), and non-fullerene acceptors (Xie and Wuerthner, 2017; Li et al., 2019) have also been utilized to construct high-performance organic–inorganic hybrid materials by passivating the inherent defects of metal oxide layers and remarkable efficiency improvements were all observed.

The reported organic defect-passivating agents can generally coordinate with ZnO due to the existence of various polar groups, such as hydroxyl groups, amino groups, carbonyl groups, and carboxylic groups. Here, we found that 2,3,5,6-tetrafluoro-7,7,8,8-tetracyanoquinodimethane (F4TCNQ) with four cyano (C=N) groups can also regulate the intrinsic property of ZnO CBLs with the assistance of coordination effects of C=N groups. In order to passivate the inherent defects originated from ZnO-based CBLs, we propose an organic–inorganic hybrid CBL composed of inorganic ZnO and organic dopant F4TCNQ and explore how the F4TCNQ doping in the CBLs affects device operation. The introduction of ZnO:F4TCNQ hybrid CBLs is able to improve the PCEs of both fullerene and non-fullerene OSCs by the remarkable increase of fill factor (FF) value. The use of F4TCNQ dopant can modify surface properties and regulate energy-level structure of ZnO-based CBLs simultaneously. Benefiting from the coordination effect of C=N groups, trap density in the ZnO film is reduced significantly, which suppresses non-geminate charge recombination in the ZnO:F4TCNQ-based OSCs. These results indicate that the construction of novel ZnO:F4TCNQ hybrid film is a promising approach in the interfacial modification of ZnO-based CBLs for the performance optimization of OSC devices.

## Results and Discussion

### Photovoltaic Performance

**Figure 1:**
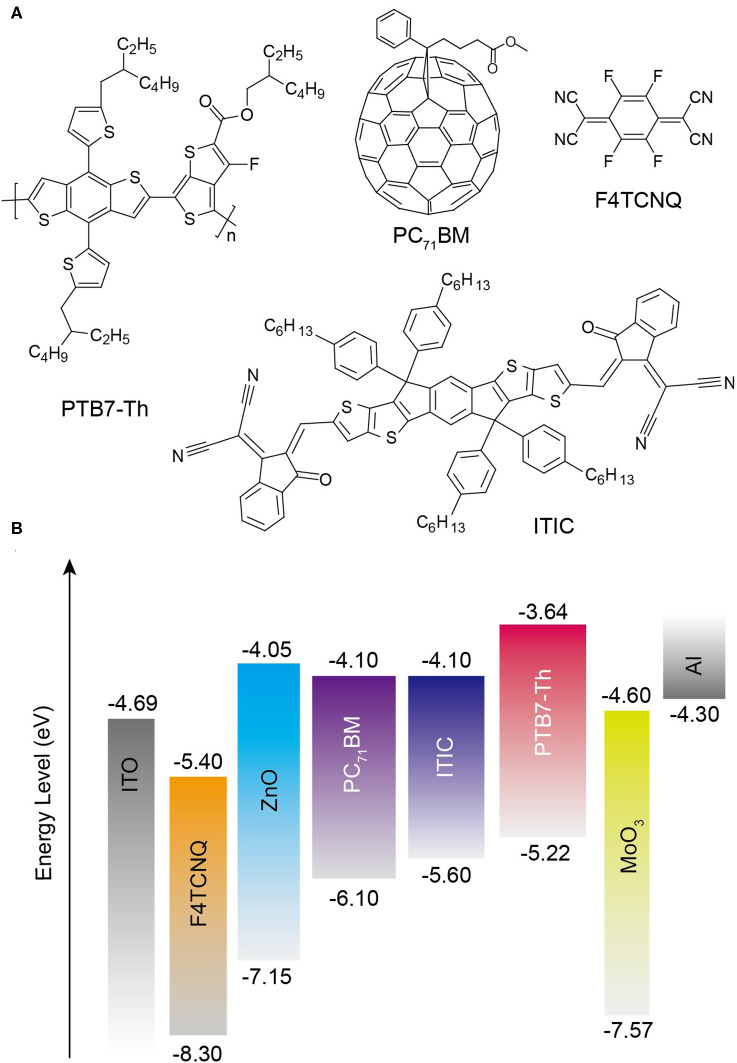
**(A)** Molecular structures of PTB7-Th, ITIC, PC_71_BM, and F4TCNQ. **(B)** A schematic device structure of the inverted OSCs and the corresponding molecular energy levels.

To passivate the ZnO defects such as oxygen vacancies and zinc dangling bonds and modify interfacial contact between metal electrode and organic photoactive layer, a novel organic–inorganic hybrid material based on ZnO and F4TCNQ was used as CBLs in both fullerene- and non-fullerene-based OSCs. Figure 1A provides the molecular structure of photoactive materials: donor poly[4,8-bis(5-(2-ethylhexyl)thiophen-2-yl)benzo[1,2-b:4,5-b′]dithiophene-co-fluorothieno[3,4-b]thiophene-2-carboxylate] (PTB7-Th), fullerene-based acceptor PC_71_BM, and non-fullerene-based acceptor 3,9-bis(2-methylene-(3-(1,1-dicyanomethylene)-indanone))-5,5,11,11-tetrakis(4-hexylphenyl)-dithieno[2,3-d:2′,3′-d′]-s-indaceno[1,2-b:5,6-b′]dithiophene) (ITIC). The molecular structure of organic dopant F4TCNQ is also supplied in Figure 1A. Meanwhile, Figure 1B shows the schematic device structure and the corresponding energy levels. Considering that sol-gel-derived ZnO generally has high density of defects, the organic dopant F4TCNQ was employed into the precursor solution to passivate the defects in both surface and bulk. Similar to the previous approach used to fabricate pure ZnO film, ZnO:F4TCNQ hybrid films with different doping concentrations were deposited by an identical sol-gel strategy (Sun et al., 2011; Li et al., 2019).

**Figure 2:**
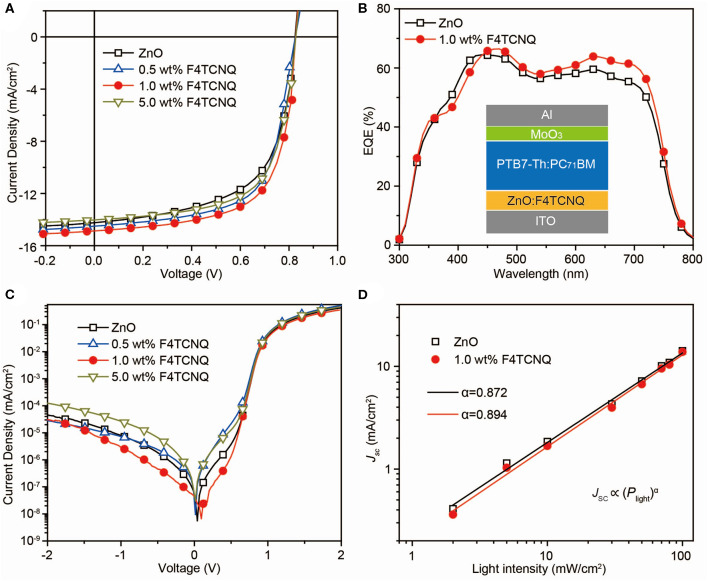
**(A)** *J–V* characteristics and **(B)** EQE spectra of the inverted PTB7-Th:PC_71_BM-based OSCs with various CBLs under illumination of AM 1.5G at 100 mW/cm^2^. Inset: Device configuration of the OSCs. **(C)** Semi-logarithmic plots of *J–V* characteristics of the OSCs with various CBLs in dark. **(D)** Measured *J*_SC_ of different CBLs dependence on light intensity.

As shown in Figure 2A, *J–V* characteristics of the inversed OSCs based on the PTB7-Th:PC_71_BM system using ZnO:F4TCNQ hybrid CBLs are investigated under AM 1.5G irradiation. The corresponding photovoltaic parameters including open-circuit voltage (*V*_OC_), short-circuit current density (*J*_SC_), FF, PCE, series resistance (*R*_S_), and shunt resistance (*R*_sh_) are summarized in Table 1. The inverted OSC device with the structure of indium tin oxide (ITO)/CBL/PTB7-Th:PC_71_BM/MoO_3_/Al is shown in Figure 2B. For the devices fabricated with ZnO:F4TCNQ hybrid CBLs, the PCE value enhances from 7.17% for pristine device to 7.55% for ZnO:0.5 wt% F4TCNQ device, 8.14% for ZnO:1.0 wt% F4TCNQ device, and 7.56% for ZnO:5.0 wt% F4TCNQ device, respectively. When organic dopant F4TCNQ with the optimal concentration of 1.0 wt% is introduced into ZnO film, the *J*_*SC*_ value increases slightly from 14.24 to 15.00 mA/cm^2^ while the FF improved from 62.2% to 67.1% significantly. The slightly enhanced external quantum efficiency (EQE) almost in the entire absorption range shown in Figure 2B is consistent with the minor variations of *J*_SC_ value as well as the slight reduction of *R*_S_. Furthermore, the ZnO:F4TCNQ hybrid thin film used as a novel CBL was also applied to PTB7-Th:ITIC-based non-fullerene OSCs. Since the LUMO levels of PC_71_BM and ITIC are quite close, the *V*_OC_ values are the same for both fullerene- and non-fullerene-based OSCs. The non-fullerene-based device exhibits improved PCE values from 6.13 to 6.96%, which also mainly contributed to the enhancement of FF values (Table 1 and Supplementary Figure 1). Therefore, the construction of ZnO:F4TCNQ hybrid CBLs could efficiently improve the final device performance of both fullerene and non-fullerene OSCs by enhancing the FF values.

**Table 1:** Performance of inverted fullerene- and non-fullerene-based OSCs with different CBLs.

**System**	**CBL**	***V*_**oc**_ (V)**	***J*_**sc**_ (mA/cm^**2**^)**	**FF (%)**	**PCE (%)**	***R*_**s**_*^a^* (Ω·cm^**2**^)**	***R*_**sh**_*^b^* (Ω·cm^**2**^)**
PTB7-Th:PC_71_BM	ZnO	0.81	14.24	62.2	7.17	8.64	508.2
	0.5 wt% F4TCNQ	0.81	14.50	66.0	7.75	5.26	662.1
	1.0 wt% F4TCNQ	0.81	15.00	67.1	8.14	4.41	913.8
	5.0 wt% F4TCNQ	0.81	14.03	66.6	7.56	6.62	839.8
PTB7-Th:ITIC	ZnO	0.81	14.35	52.8	6.13	11.5	415.4
	0.5 wt% F4TCNQ	0.81	14.48	57.1	6.69	12.3	475.7
	1.0 wt% F4TCNQ	0.81	14.75	58.3	6.96	9.44	549.5
	5.0 wt% F4TCNQ	0.81	14.87	57.4	6.91	9.71	484.5

a Series resistance (R_s_) and

b *Shunt resistance (R_sh_)*.

The *J–V* characteristics of inverted OSCs fabricated with ZnO:F4TCNQ hybrid CBLs in the dark condition were measured to investigate the inherent electrical properties, as shown in Figure 2C. Compared to the pristine ZnO-based device, the introduction of 1.0 wt% F4TCNQ leads to a significant reduction of leakage current, which demonstrates the efficient hole blocking capability of the ZnO:F4TCNQ hybrid film. On the contrary, the leakage current becomes strengthened with the further increase of F4TCNQ content (5 wt%). To investigate the charge recombination dynamics in the PTB7-Th:PC_71_BM system, the dependence of *J*_SC_ on light intensity (*P*_light_) was also conducted under the optimal F4TCNQ dopant content (Figure 2D). Generally, the dependence of *J*_SC_ on *P*_light_ can be expressed as *J*_SC_∝(*P*_light_)^α^ (Zhao et al., 2017). Figure 2D shows that the fitted slope (α = 0.897) of the ZnO:F4TCNQ-based device is much closer to 1, thus demonstrating the beneficial effect of the introduced F4TCNQ dopant on the reduction of bimolecular recombination process. In addition, the significantly enhanced *R*_sh_ from 508.2 to 913.8 Ω·cm^2^ for the ZnO:F4TCNQ-based device also indicates a weakened carrier recombination at the ZnO/photoactive layer interfaces (Yin et al., 2013). Therefore, the significantly enlarged *R*_sh_ and the restrained leakage current suggest that the introduced F4TCNQ component plays a significant role in optimizing the interfacial contact between different functional layers, thus facilitating the remarkable enhancement of FF value. When F4TCNQ was doped into ZnO film, a similar *R*_sh_ enhanced rule is observed for non-fullerene OSCs. Consequently, organic dopant F4TCNQ in both fullerene and non-fullerene systems is capable of suppressing charge recombination events, thus significantly enhancing the FF and PCE values of OSCs.

### Properties of ZnO:F4TCNQ Hybrid Thin Films

**Figure 3:**
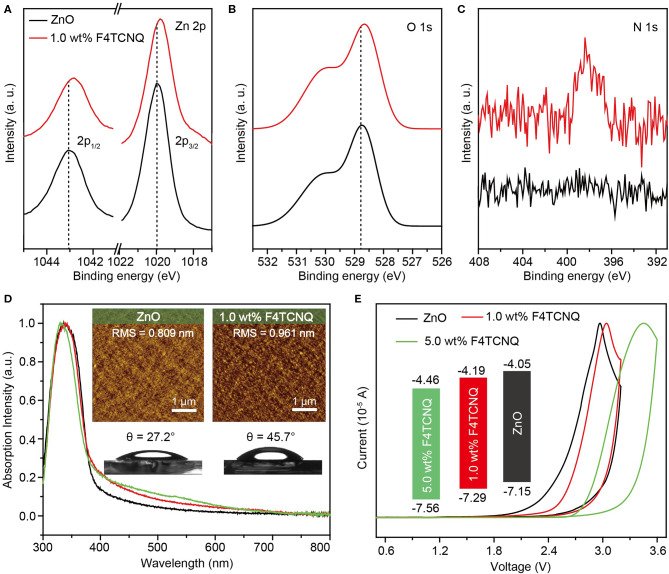
**(A)** Zn 2p, **(B)** O 1s, and **(C)** N 1s high-resolution XPS spectra, **(D)** UV-vis absorption spectra, AFM height images (5 × 5 μm) and the contact angle images (inset), **(E)** CV measurements and energy level diagram (inset) of various ZnO-based CBLs.

X-ray photoelectron spectroscopy (XPS) measurements were conducted to explore the effect of F4TCNQ dopant on the formation of ZnO-based CBLs (Supplementary Figure 2). The high-resolution XPS spectra of Zn 2p_1/2_, Zn 2p_3/2_, O 1s, and N 1s are shown in Figures 3A–C. When 1.0 wt% F4TCNQ was doped, the peaks of Zn 2p_1/2_ (~1043.0 eV) and Zn 2p_3/2_ (~1019.9 eV) shift toward lower binding energy by ~0.2 and ~0.1 eV, respectively. Meanwhile, the main peak of O 1s (~528.8 eV) corresponding to O atoms in ZnO matrix also shifts toward lower binding energy by ~0.1 eV. The peak shifts shown in Figures 3A,B indicate that more Zn atoms are bound to O atoms and the oxygen-deficient defects originated from hydroxyl groups or carboxylate groups are passivated in the ZnO:F4TCNQ hybrid film (Sun et al., 2011; Li et al., 2017). In addition, compared to the pure ZnO film, the appearance of N 1s peak for ZnO:F4TCNQ hybrid film further demonstrates that the organic dopant F4TCNQ has been incorporated into ZnO film.

Cyclic voltammetry (CV) measurements combined with UV-vis absorption spectra were performed to investigate the impact of introduced F4TCNQ on the energy levels of ZnO-based CBLs. As shown in Figure 3D, the employed F4TCNQ dopant exhibits a marginal impact on the optical properties of ZnO film and thus it can be approximately considered that the band gap of ZnO-based CBLs remains unchanged when a small amount of F4TCNQ molecules are added into ZnO precursor solution. Meanwhile, the highest occupied molecular orbital (HOMO) levels of ZnO and ZnO:F4TCNQ hybrid films were characterized by CV measurement (Figure 3E). The HOMO level of pure ZnO calculated from the onset potential of oxidation process is found to be −7.15 eV. While, the onset potential of ZnO:1.0 wt% F4TCNQ oxidation process shifts toward larger potential position and the corresponding HOMO level is calculated to be −7.29 eV. When the content of F4TCNQ is further raised to 5.0 wt%, the HOMO level of ZnO:F4TCNQ hybrid CBL reduces to −7.56 eV. According to the UV-vis absorption spectra and CV measurements of ZnO-based CBLs, the corresponding lowest unoccupied molecular orbital (LUMO) level can be obtained, as shown in the inset of Figure 3E. The optimized energy level structure by employing F4TCNQ dopant can lower the charge barrier of electron collection and thus be expected to facilitate the acquirement of high-performance OSCs.

In addition, we also performed atomic force microscopy (AFM) and contact angle (θ) measurement to reveal the influence of F4TCNQ dopant on the surface properties of ZnO-based CBLs, as shown in the inset of Figure 3D. Compared to the pure ZnO film with the roughness of 0.809 nm, the ZnO:1.0 wt% F4TCNQ hybrid film exhibits similar film roughness (0.961 nm). When the content of F4TCNQ reaches 5.0 wt%, the roughness is further increased to more than 1 nm and the remarkable aggregates can be observed at the film surface (Supplementary Figure 3). Therefore, the content of F4TCNQ dopant should be adjusted cautiously to fabricate the ZnO-based hybrid CBLs with excellent interfacial properties. Besides, the contact angle (θ) measurement provides the hydrophobic property of ZnO-based CBLs (the inset of Figure 3D). Due to the excellent hydrophobicity of F4TCNQ itself, the constructed ZnO:F4TCNQ hybrid CBL exhibits an enhanced hydrophobic property with the θ of 45.7°, which can perform a beneficial role in optimizing interfacial contact between CBL and organic photoactive layer (Hau et al., 2008; Liao et al., 2013). Furthermore, the conductivity measurements of the ZnO and ZnO:F4TCNQ films were also conducted (Supplementary Table 1). It was found that the ZnO:F4TCNQ film slightly decreases from 2.06 × 10^−3^ to 1.56 × 10^−3^ S/cm, indicating that the electron transfer from ZnO to the organic electron-acceptor molecule is not very efficient (Hewlett and McLachlan, 2016). This variation further demonstrates that the primary function of F4TCNQ is to optimize the interfacial contact rather than the inherent carrier transport properties of ZnO.

### Trap-Filling Mechanism

**Figure 4:**
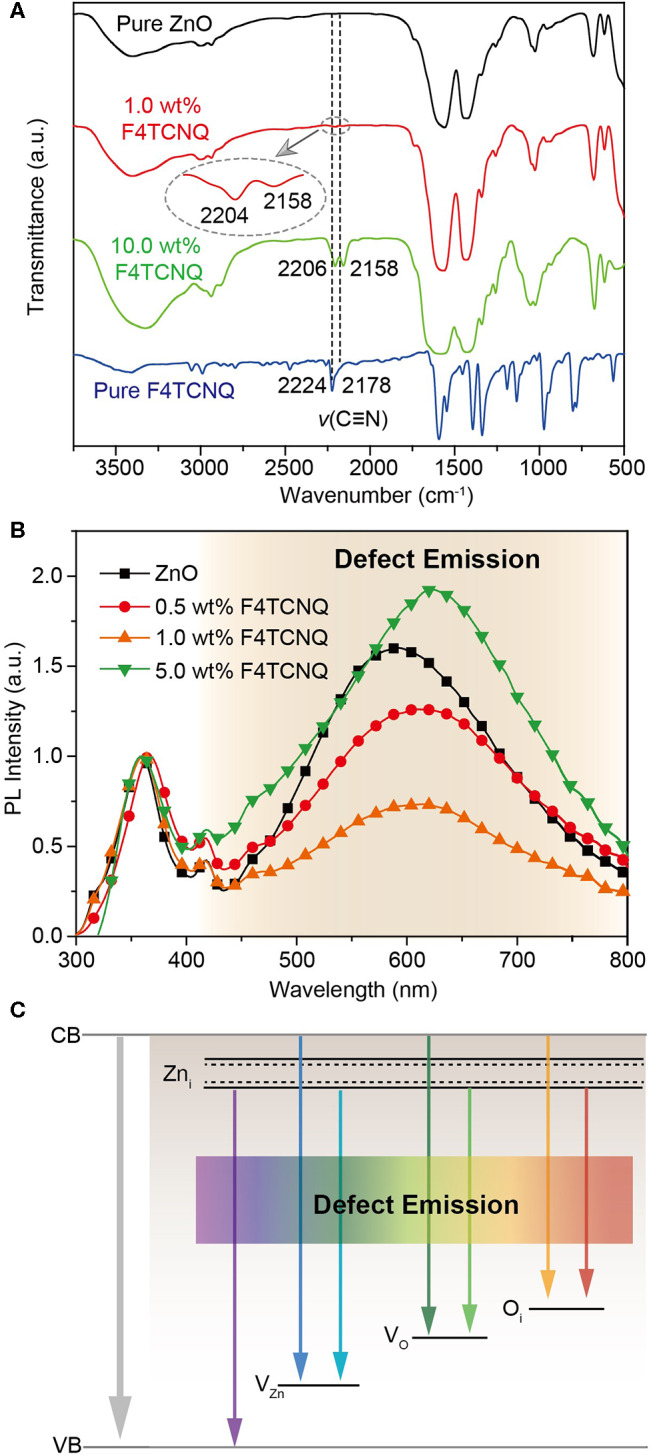
**(A)** FTIR spectra of pure ZnO, pure F4TCNQ, and ZnO:x wt% F4TCNQ films (*x* = 1.0 and 10.0). **(B)** PL spectra of pristine ZnO and ZnO:x wt% F4TCNQ films (*x* = 0.5, 1.0, and 5.0) on top of the quartz substrates. **(C)** Energy level diagram of native defect states in sol-gel-derived ZnO.

Fourier transform infrared (FTIR) spectra are conducted to investigate the intermolecular interaction between F4TCNQ and ZnO (Figure 4A). In the pure F4TCNQ film, the stretching vibrations related to the four C=N groups appear at 2,224 and 2,178 cm^−1^, respectively (Kato et al., 1991), while there is no characteristic peak that appeared at around 2,200 cm^−1^ for pure ZnO film. As soon as 1 wt% F4TCNQ was doped into ZnO, the two characteristic peaks of C=N stretching vibration shift to 2,204 and 2,158 cm^−1^, respectively. Considering that the peak signal of C=N stretching vibration is relatively weak in the ZnO:1.0 wt% F4TCNQ film, the sample of ZnO:10.0 wt% F4TCNQ film was also detected and the same changes have been observed. The red shift of C=N stretching vibration peaks indicates that the C=N groups in F4TCNQ molecules coordinate with ZnO (Zhou et al., 2016). Benefiting from the coordination effects between F4TCNQ and ZnO, the passivation of various defects that existed in the ZnO matrix can be anticipated.

According to the previous reports, the photoluminescence (PL) properties of ZnO are very sensitive to the defects inside (Chen et al., 2012; Mishra et al., 2019). As shown in Figure 4B, the narrow emission peak at 363 nm is attributed to the band-to-band emission of ZnO, while the broad emission one at around 600 nm stems from the defect states in ZnO matrix (Mishra et al., 2019). The broad emissions correspond to various defects, e.g., Zn_i_ (violet), V_Zn_ (blue), V_O_ (green), and O_i_ (yellow, red), and the qualitative energy levels of these defect states in sol-gel ZnO are shown in Figure 4C. It should be noticed that the peak intensity of defect emission is remarkably stronger than that of ZnO emission for the pristine ZnO film, which demonstrates the existence of a large amount of defect states in the sol-gel-derived ZnO film. Upon introducing a few amount of F4TCNQ (0.5 wt% and 1.0 wt%), the broadband defect emission peak is effectively weakened, indicating a significant reduction of defect states. On the contrary, when the content of F4TCNQ is further increased to 5 wt%, the condition of defect states deteriorates and the emission peak corresponding to the defect states becomes even stronger. We speculate that excessive doping of F4TCNQ induces the formation of new defects in the ZnO-based CBLs. To exclude the impact of emission interference from F4TCNQ itself, the PL spectrum of the pure F4TCNQ film was also measured and the corresponding fluorescence signal in the range of visible region was found to be very weak (Supplementary Figure 4). The results indicate that an appropriate amount of F4TCNQ dopant can conduce to the construction of an excellent ZnO thin film with less defect states. In addition, the time-resolved PL spectra of pure PC_71_BM layers were also measured and the exciton lifetime of PC_71_BM on the ITO/ZnO:F4TCNQ substrate was found to be slightly longer than that on the ITO/ZnO substrate. This confirms that the photo-generated excitons prefer to transfer to the ZnO:F4TCNQ layer, leading to reduced charge recombination (Supplementary Figure 5).

Trap-assisted recombination that happened in the ZnO film can be regarded as a two-step process (Supplementary Figure 6); i.e., a hole is first trapped within the band gap; then, an oppositely charged electron finds the trapped hole and thus recombines with it (Proctor et al., 2013). Generally, the holes should be transported through the donor phase to the anode electrode when the photogenerated carriers have formed in the photoactive layer. However, a mass of defects generated in the ZnO film may induce hole transfer from the donor component to the ZnO CBL. When the electrons are collected through ZnO-based CBLs, they may enter the trap states with positively charged species and trap-assisted recombination process happens. According to the previous reports, the trap-assisted recombination rate is ultimately determined by the amount of trap sites and how quickly the free carrier can find the trapped carrier (Kirchartz et al., 2011; Proctor et al., 2013). As soon as F4TCNQ dopants were employed, the introduction of F4TCNQ dopants will fill the defects where traps reside, thus rendering them electronically inert (Yan et al., 2016). Based on the trap-filling strategy, the photogenerated holes will be blocked from entering trap states and thus the probability of trap-assisted recombination can be reduced efficiently during carrier transport process.

## Conclusions

In conclusion, the ZnO:F4TCNQ hybrid films with good solution processability, tunable energy levels, and reduced defects have been developed as a new class of CBLs for both fullerene- and non-fullerene-based OSCs. While adding F4TCNQ into ZnO film, remarkable increases in FF were observed, leading to an obvious enhancement in overall device performance. The surface property and energy levels of ZnO-based CBLs can be regulated efficiently by the doping of organic acceptor. Owing to the coordination effects between cyano groups and ZnO, the introduced F4TCNQ contributes to suppressing the non-geminate recombination by filling the ZnO defects, thus allowing for efficient charge transport and extraction in ZnO-based CBLs. Consequently, the simple trap-filling strategy by organic dopants provides an efficient approach in adjusting electrical properties of interfacial layers, which is important for constructing high-performance CBLs of inverted OSCs.

## Experimental

### Materials

PTB7-Th, PC_71_BM, and ITIC were purchased from Luminescence Technology Corp. O-dichlorobenzene (ODCB) and chlorobenzene (CB) were purchased from Aladdin Corp. Zinc acetate dihydrate [Zn(CH_3_COO)_2_•2H_2_O, 98%], ethanolamine (NH_2_CH_2_CH_2_OH, 99.5%), and 2-methoxyethanol (CH_3_OCH_2_CH_2_OH, 99.8%) were purchased from Acros. 2,3,5,6-Tetrafluoro-7,7,8,8-tetracyanoquinodimethane (F4TCNQ) was provided by Sigma-Aldrich. Molybdenum oxide (MoO_3_) was provided by Shanghai Han Feng Chemical Corp. All chemicals were used as obtained.

### Preparation of CBLs

Sol-gel-derived ZnO CBLs were prepared using zinc acetate in 2-methoxyethanol:ethanolamine as a precursor solution by spin-coating onto the ITO substrate followed by thermal annealing at 200°C for 30 min according to the reference (Sun et al., 2011). As for the ZnO:F4TCNQ hybrid CBLs, the ZnO:F4TCNQ precursor solutions were prepared by mixing with different contents of F4TCNQ (0.5, 1.0, and 5.0 wt%) and dissolving in a mixture of 2-methoxyethanol as the solvent and ethanolamine as the stabilizer under sufficient stirring. After the spin-coating process has been completed, the hybrid film was also thermal-annealed at 200°C for 30 min.

### Device Fabrication

The device structure was ITO/CBL/PTB7-Th:PC_71_BM/MoO_3_/Al. Firstly, the CBLs (~30 nm) were spin-coated on top of the precleaned ITO substrate, which was treated with deionized water, acetone, and ethanol in an ultrasonic bath followed by ultraviolet (UV)-ozone treating for 15 min. A blend of PTB7-Th:PC_71_BM with the mass ratio of 4:7 in ODCB solvent was spin-coated on the surface of CBLs in the N_2_ glovebox. The thickness of the active layer was controlled to be around 100 nm. Then, MoO_3_ film (10 nm) as the anode interfacial layer was thermally evaporated on the active layer, followed by the thermal evaporation of Al (~100 nm). The device of ITO/ZnO/PTB7-Th:ITIC/MoO_3_/Al was prepared by adopting a similar approach. For the non-fullerene-based photoactive layer, a blend of PTB7-Th:ITIC with the mass ratio of 1:1.2 in CB solvent was spin-coated. The thickness of the active layer was also controlled to be around 100 nm. These OSCs, made on ITO glass substrate, have an active area of 0.09 cm^2^.

### Measurements and Characterization

Current density-voltage (*J–V*) characteristics of OSCs, which were detected under ambient situation, were recorded by Keithley-2400 digital source meter. SAN-EI electric solar simulator (XES-50S1) under simulated AM 1.5 illumination (100 mW/cm^2^) was used for solar cell characterization. The EQE measurements of the PSCs were carried out with QE-R system (Enli Technology Co., Ltd.). The surface morphology of the CBLs was characterized by atomic force microscopy (AFM, FM-Nanoview 1000, FSM-Precision Co., Ltd.) in taping mode. Contact angle characterizations were carried out on pure ZnO layer, and ZnO:F4TCNQ layer using droplet shape analyzer (KRÜSS GmbH Germany). X-ray photoelectron spectroscopy (XPS) experiments were carried out with ESCALAB 250 system. The measurement chamber was equipped with a monochromatic Al Kγ X-ray source (hγ = 1486.6 eV). Ultraviolet-visible (UV-Vis) absorption spectra were measured on a lambda 35 PerkinElmer UV-Vis spectrophotometer. Photoluminescence (PL) spectra and time-resolved PL measurements were performed on an Edinburgh FLS980 fluorescence at room temperature. CV measurement was performed to estimate the HOMO from the onset potential of the electrochemical oxidation. The CV measurement was carried out on a CHI660E system in a three-electrode cell with a working electrode, a reference electrode, and a counter electrode in an acetonitrile solution of Bu_4_NPF_6_ (0.1 M). Fourier transform infrared (FTIR) spectroscopic analysis was performed using FT-IR spectrometer (PerkinElmer). The electrical conductivity was measured by Physical Property Measurement System (Quantum Design).

## Data Availability Statement

All relevant data is contained within the article. The raw data supporting the conclusions of this article will be made available by the authors, without undue reservation, to any qualified researcher.

## Author Contributions

ML and JL conceived the ideas and performed the device fabrication. LY and YZ conducted the XPS, UV-vis, and CV measurements. YD conducted the AFM and FTIR measurements. RC and WH supervised the research and contributed to manuscript preparation.

## Conflict of Interest

The authors declare that the research was conducted in the absence of any commercial or financial relationships that could be construed as a potential conflict of interest.
