# Supplementary material for: Trap-Filling of ZnO Buffer Layer for Improved Efficiencies of Organic Solar Cells
Source: Front Chem. 2020 May 26;8:399. doi: 10.3389/fchem.2020.00399 (PMC7264381; doi:10.3389/fchem.2020.00399)
Supplement: Supplementary file 1 [file Table_1.DOCX]

Supplementary Material

**
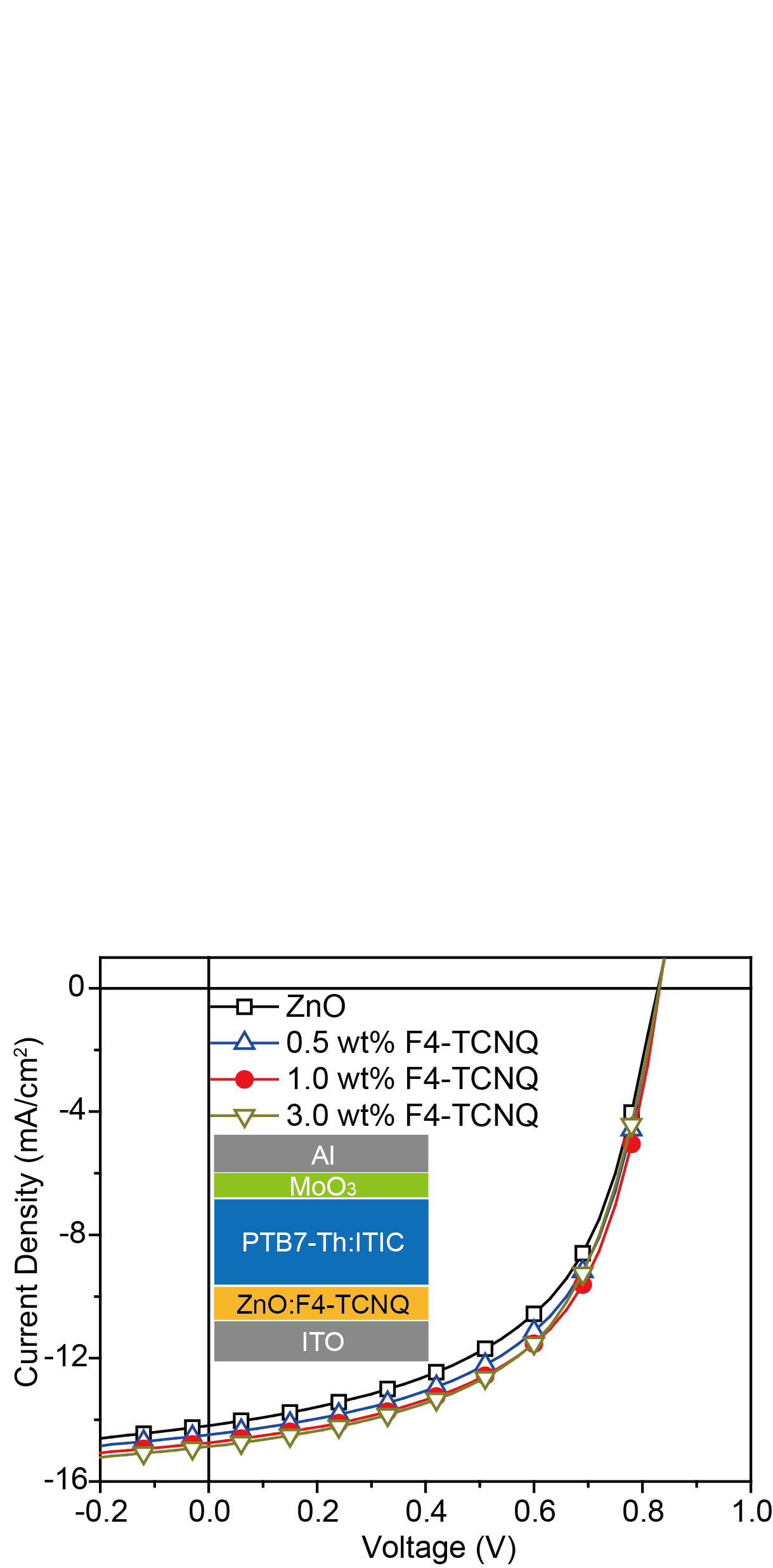
**

**Supplementary Figure 1.** *J-V* characteristics of the inverted PTB7-Th:ITIC solar cells with various CBLs under illumination of AM 1.5G at 100 mW/cm^2^. Inset: Device configuration of the ZnO:F4TCNQ-based non-fullerene OSCs.

**
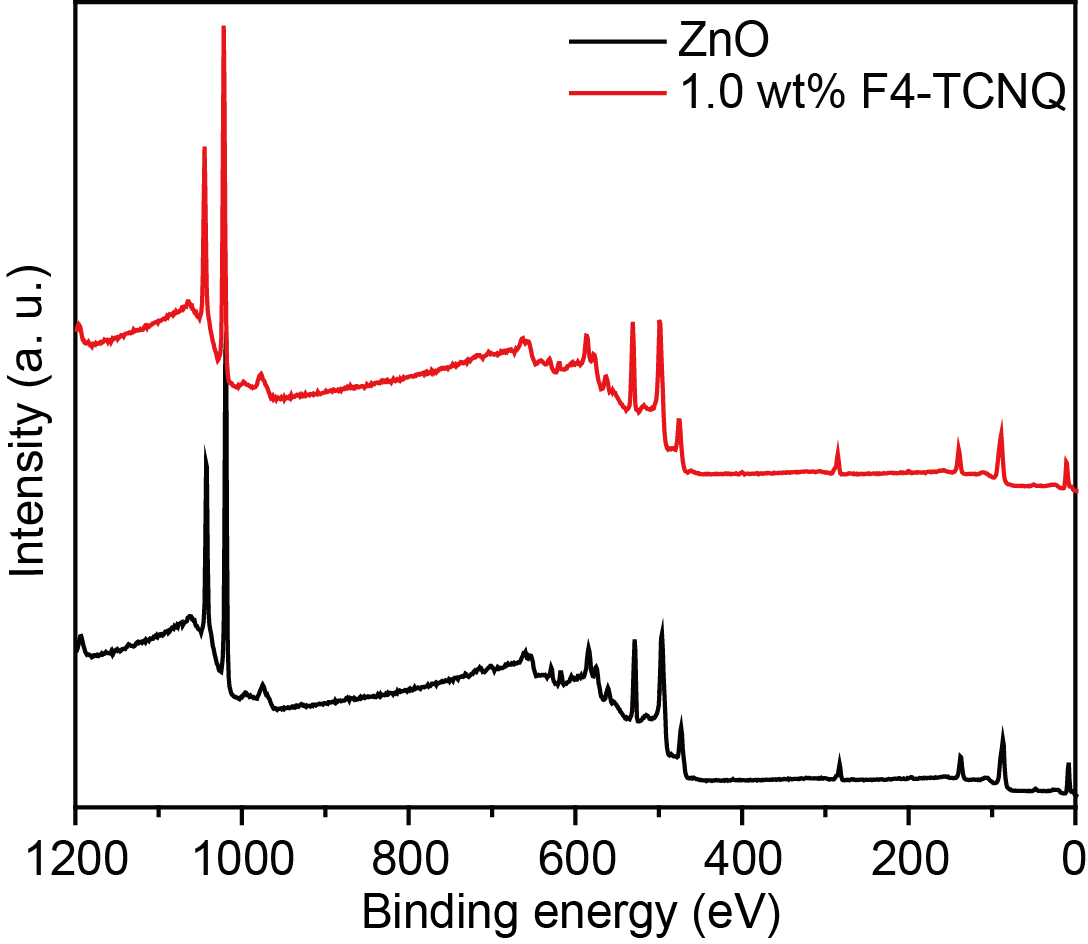
**

**Supplementary Figure 2.** XPS spectra of ZnO and ZnO:1.0 wt% F4TCNQ films.

**
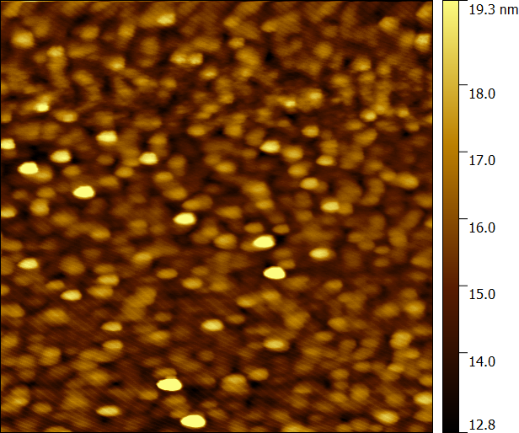
**

**Supplementary Figure 3.** AFM height images (5 μm×5 μm) of ZnO:5 wt% F4TCNQ film.

**
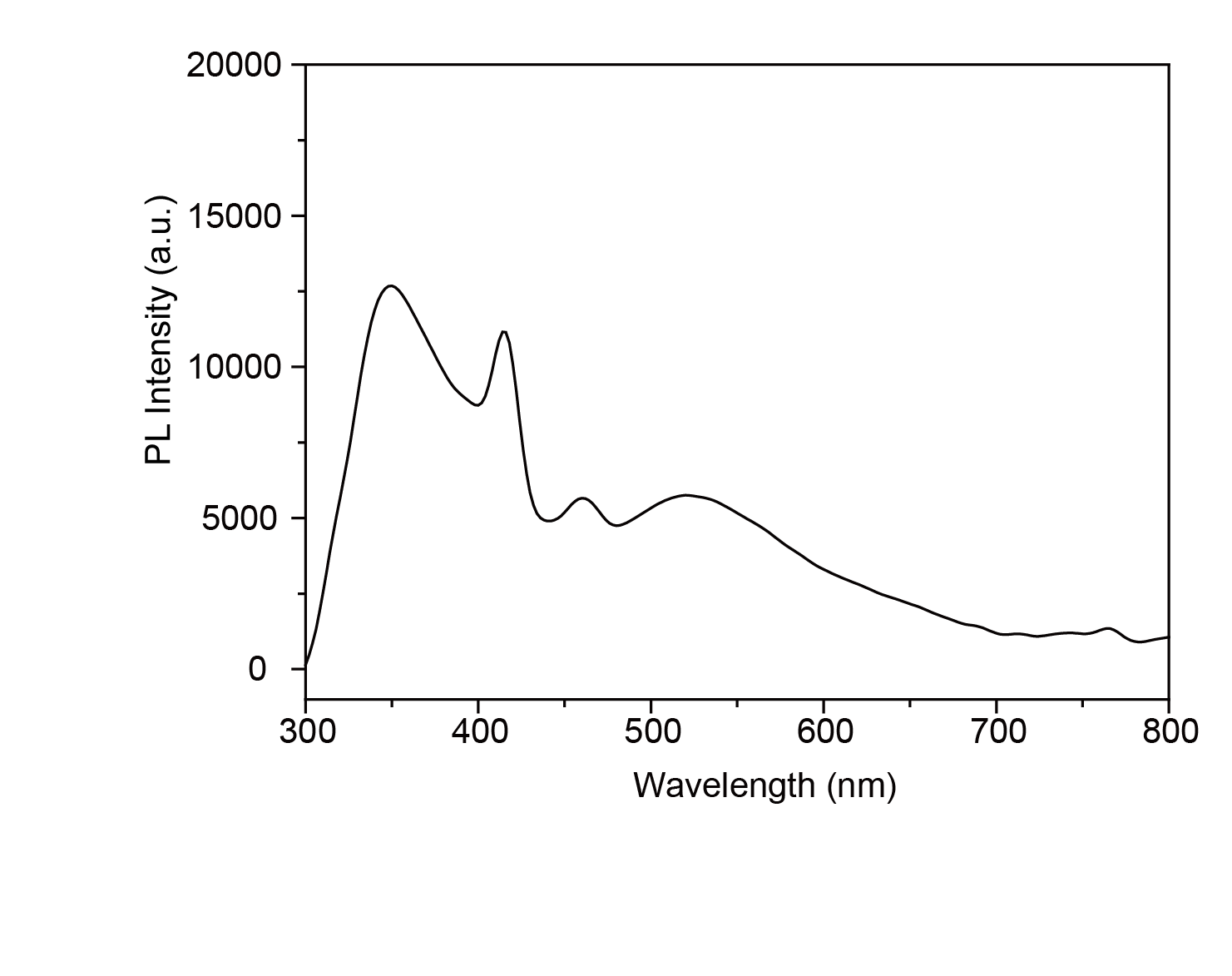
**

**Supplementary Figure 4.** PL spectrum of pure F4TCNQ film on top of the quartz substrate.


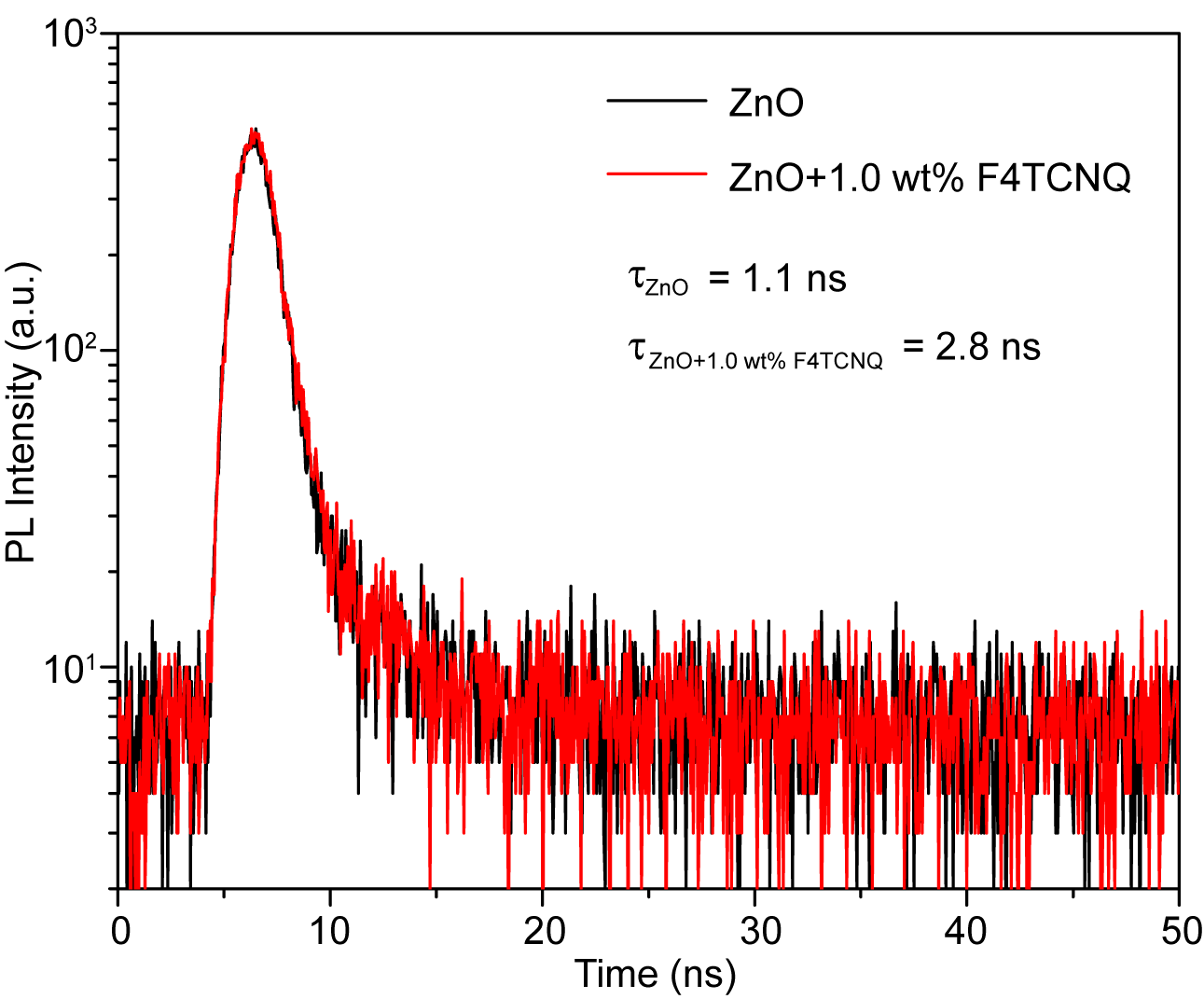


**Supplementary Figure 5.** Time resolved PL spectra of PC_71_BM layers on ITO/ZnO and ITO/ZnO:F4TCNQ substrates.


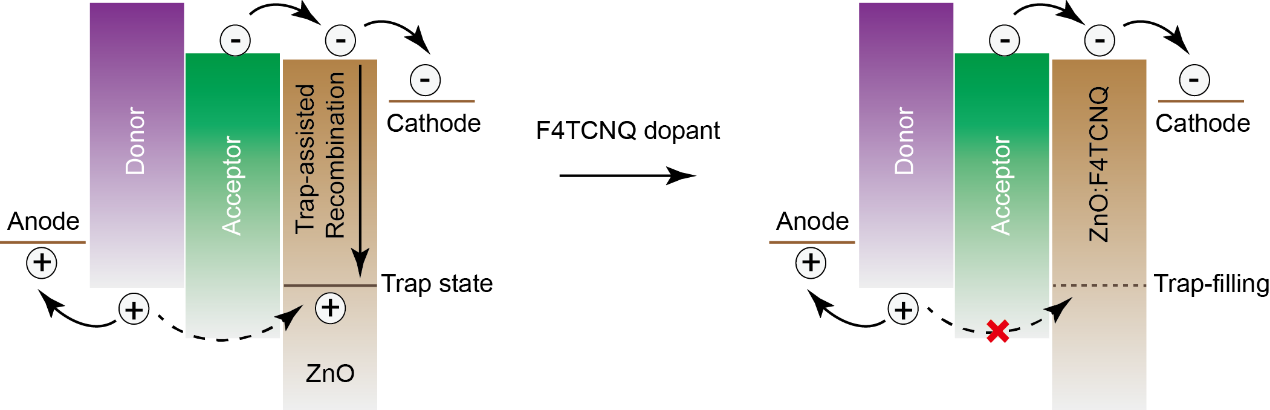


**Supplementary Figure 6.** The proposed models of trap-assisted recombination and trap-filling in the ZnO-based CBLs.

**Supplementary Table 1.** Conductivity measurement of ZnO-based CBLs

| Sample | Type of charges | Surface charge density (cm^-2^) | Bulk charge density (cm^-3^) | Resistivity (Ω‧cm) | Conductivity (S/cm) |
| --- | --- | --- | --- | --- | --- |
| ZnO | n | 5.88704×10^8^ | 1.96235×10^14^ | 484.284 | 2.06×10^-3^ |
| ZnO: 1.0 wt% F4TCNQ | n | 5.49029×10^8^ | 1.83010×10^14^ | 640.735 | 1.56×10^-3^ |
| ZnO: 10.0 wt% F4TCNQ | n | 5.20963×10^8^ | 1.73654×10^14^ | 765.661 | 1.31×10^-3^ |
